# High lethality rate of carbapenem-resistant *Acinetobacter baumannii* in Intensive Care Units of a Brazilian hospital: An epidemiologic surveillance study

**DOI:** 10.1590/0037-8682-0529-2021

**Published:** 2022-04-29

**Authors:** Mariana Neri Lucas Kurihara, Romário Oliveira de Sales, Késia Esther da Silva, Gerlaine Damasceno Silva, Maria Carolina Tazinazzo Mansano, Fuad Fayez Mahmoud, Simone Simionatto

**Affiliations:** 1 Universidade Federal da Grande Dourados, Laboratório de Pesquisa em Ciências da Saúde, Dourados, MS, Brasil.; 2 Stanford University, Division of Infectious Diseases and Geographic Medicine, Stanford, CA, USA.; 3 Universidade Federal da Grande Dourados, Hospital Universitário de Dourados, Dourados, MS, Brasil.

**Keywords:** Acinetobacter baumannii, Carbapenem-resistant, Epidemiology, Surveillance, Lethality

## Abstract

**Background:**

Carbapenem-resistant *Acinetobacter baumannii* (CRAB) is a growing threat to public health.

**Methods:**

A 3-year retrospective study was conducted to evaluate the prevalence and lethality of multidrug-resistant (MDR) *A. baumannii* isolated from Brazilian patients.

**Results:**

In this study, 219 *Acinetobacter baumannii* isolates were identified, of which 70.8% (155/219) were isolated from patients hospitalized in intensive care units. Of these, 57.4% (n = 89/155) were assessed, of which 92.1% (82/89) were carbapenem-resistant, and 49 were classified as infected. The lethality rate was 79.6% (39/49).

**Conclusions:**

We highlight the need of an effective epidemiological surveillance measure to contain the dissemination of CRAB in the hospital environment.

*Acinetobacter baumannii* is widely recognized as an opportunistic pathogen in medical clinics, with the ability to colonize and infect hospital patients or discharged patients^1^. It also increases the risk of disseminating multidrug-resistant (MDR) microorganisms and interferes with the safety and quality of inpatient treatment^2^. The widespread and prolonged use of broad-spectrum antibiotics favors selective pressure, contributing to the emergence of MDR *A. baumannii* and restricting treatment options for infected patients. The excessive use of colistin as a last resort for therapy may have contributed to the increased number of *A. baumannii* resistant to colistin^3^. In 2017, *A. baumannii* was considered an alarming global health issue by the World Health Organization (WHO), which established its spread control as a priority^4^. Epidemiological studies on resistant bacteria may help formulate appropriate intervention plans for each hospital, thus reducing the spread of the pathogen in the nosocomial environment. In this 3-year retrospective study, we investigated the prevalence and lethality of MDR *A. baumannii* isolates from patients admitted to a Brazilian tertiary hospital. 

This retrospective study was conducted at a tertiary hospital in Dourados, Brazil, from January 2015 to December 2017. Patients with *A. baumannii* isolated from clinical cultures were included in the study. Records with incomplete data, patients transferred to other hospitals prior to discharge from the intensive care unit (ICU), or were not monitored for loss of data or incorrect records, as well as records of the same patient with a different clinical source of infection, were excluded (Figure 1). For infectivity and lethality rates, we selected colonization or infection cases from all patients admitted to the ICU with *A. baumannii* isolates^5^ *.* This study was approved by the Research Ethics Committee of Universidade Federal da Grande Dourados (no. 877.292/2014). 

**FIGURE 1:**
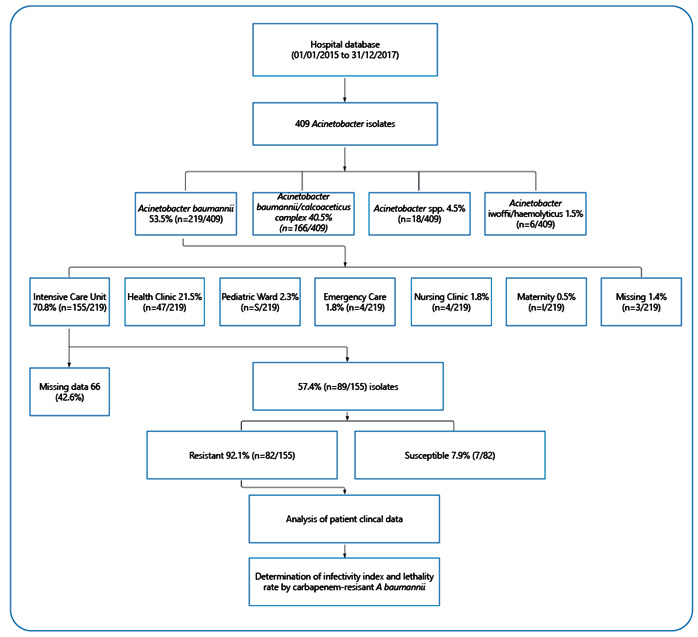
Flowchart of study design.

The clinical data of the patients admitted to the hospital were retrospectively analyzed. The following data were recorded: demographic characteristics, medical history, comorbidities, source of isolation, invasive procedures, mechanical ventilation, total parenteral nutrition, urinary catheter, drainage tube, nasogastric tube, tracheal intubation, treatment with immunosuppressive drugs, and source of infection. Clinical outcome (recovery/death) data were assessed. Septic shock was defined as sepsis associated with organ dysfunction accompanied by persistent hypotension after volume replacement (Table 1). The Centers for Disease Control and Prevention (CDC) definitions for colonization and infection were used to determine the association of isolated organisms. Colonization was defined as the presence of bacteria on a patient’s skin or mucous membranes, but dissociated from signs or symptoms of infectious disease. Infection was defined by medical diagnosis according to the clinical criteria associated with the use of antimicrobial therapy and isolation of resistant *A. baumannii* strain^6^. The infectivity index was calculated using the variation in the incidence of hospital infections caused by *A. baumannii.* An epidemic or outbreak was defined as a rate above the upper control limit, and the alert period was defined as a rate above the upper alert threshold^7^. The lethality rate was calculated using the following equation: (number of deaths due to an infection in a given area and period / total number of people infected in the same area and period) × 100.

**TABLE 1:** Carbapenem-resistant *A. baumannii* (CRAB) isolates from patients admitted to the ICU of a tertiary hospital in Dourados/MS.

Variables	Validated cases	Nº		
		2015	2016	2017
Records analyzed	89 (100%)	37 (41.6%)	22 (24.7%)	30 (33,7%)
**Resistance profile**				
Susceptible	7 (7.9%)	5 (13.5%)	2 (9.1%)	-
Resistant	82 (92.1%)	32 (86.5%)	20 (90.9%)	30 (100%)
**Sex**				
Man	48 (58.5%)	15 (46.9%)	14 (70%)	19 (63,3%)
Woman	34 (41.5%)	17 (53.1%)	6 (30%)	11 (36,7%)
**Age**				
More than 60 years	43 (52.4%)	18 (56.2%)	9 (45%)	16 (53,3%)
Among 18 to 59 years	34 (41.5%)	14 (43.8%)	8 (40%)	12 (40%)
Among 0 to 18 years	5 (6,1%)	-	3 (15%)	2 (6,7%)
**Ward**				
Adult Intensive Care Unit A	35 (42.7%)	14 (43.7%)	10 (50%)	11 (36,6%)
Adult Intensive Care Unit B	42 (51.2%)	18 (56.3%)	7 (35%)	17 (56,7%)
Neonatal Intensive Care Unit	2 (2.4%)	-	2 (10%)	-
Pediatric Intensive Care Unit	3 (3.7%)	-	1 (5%)	2 (6,7%)
**Isolated material**				
Tracheal aspirate	39 (47.6%)	14 (43.8%)	13 (65%)	12 (40%)
Catheter tip	7 (8.6%)	1 (3.1%)	3 (15%)	3 (10%)
Swab	26 (31.7%)	15 (46.9%)	-	11(36.7%)
Blood culture	6 (7.3%)	1 (3.1%)	2 (10%)	3 (10%)
Uroculture	2 (2.4%)	-	1 (5%)	1 (3.3%)
Eschar	1 (1.2%)	1 (3.1%)	-	-
Vaginal secretion	1 (1.2%)	-	1 (5%)	-
**Comorbity**				
Pneumonia	52 (63.4%)	21 (65.7%)	15 (75%)	16 (53.3%)
Systemic arterial hypertension	36 (43.9%)	17 (53.1%)	7 (35%)	12 (40%)
Ischemic vascular stroke	12 (14.6%)	7 (21.9%)	3 (15%)	2 (6.7%)
HIV-AIDS	7 (8.5%)	2 (6.2%)	2 (10%)	3 (10%)
Diabetes mellitus	35 (42.7%)	14 (43.8%)	7 (35%)	14 (46.7%)
Sepsis	23 (28%)	7 (21.9%)	6 (30%)	10 (33.3%)
Pulmonary sepsis	20 (24.4%)	10 (31.3%)	4 (20%)	6 (20%)
H1N1 influenza	4 (4.9%)	1 (3.1%)	3 (15%)	-
Tuberculosis	3 (3.7%)	-	-	3 (10%)
Acute renal failure	28 (34.1%)	13 (40.6%)	5 (25%)	10 (33.3%)
Congestive heart failure	7 (8.5%)	5 (15.6%)	1 (5%)	1 (3.3%)
COPD	10 (12.2%)	5 (15.6%)	3 (15%)	2 (6.7%)
Acute respiratory insufficiency	17 (20.7%)	6 (18.8%)	3 (15%)	8 (26.7%)
**Invasive Procedures**				
Mechanical ventilation	71 (86.6%)	26 (81.2%)	20 (100%)	25 (83.3%)
NP/VDP	72 (87.8%)	27 (84.4%)	18 (90%)	27 (90%)
Tracheostomy	30 (36.6%)	10 (31.3%)	7 (35%)	13 (43.3%)
Central venous access	46 (56.1%)	18 (56.3%)	12 (60%)	16 (53.3%)
Surgery	17 (20.7%)	3 (9.4%)	10 (50%)	4 (13.3%)
Hemodialysis	21 (25.6%)	5 (15.6%)	5 (25%)	11 (36.7%)

**ICU A:** intensive care unit A; **ICU B:** intensive care unit B; **HIV-AIDS:** acquired immunodeficiency syndrome; **NP/VDP:** nasoenteric probe/vesical delay probe; **COPD:** chronic obstructive pulmonary disease.

The study period included the outbreak investigation phase in 2015, the intervention phase in 2016, and the follow-up phase in 2017 at the ICUs. This investigation occurred after an increase in the incidence of carbapenem-resistant *Acinetobacter baumannii* (CRAB) infections. Intervention at the hospital occurred in June 2016, which established neonate contact isolation during the entire hospitalization period within the neonatal ICU, regardless of the culture outcome. Surveillance cultures were conducted from all patients hospitalized for more than 48 h in different wards, especially with the previous hospitalization. Limited sharing of patient equipment, hand hygiene promotion in the ICUs, general environmental cleaning, and disinfection with the biguanide-based solution of reusable medical equipment were properly executed. Cleaning of all surfaces, including walls, floors, furniture, ceilings, windows, and medical equipment, was intensified. The follow-up occurred with analysis throughout 2017 after implementing the hospital’s intervention plan to verify the effectiveness of the applied method, as the pathogen can survive and persist on surfaces, causing hospital outbreaks^8^. 

Bacterial species were identified and tested for antimicrobial susceptibility using a BD Phoenix^TM^ system (BD Biosciences, USA). The minimal inhibitory concentrations (MICs) were determined using the broth microdilution method following the Clinical and Laboratory Standards Institute Guidelines (CLSI)^9^. The antimicrobials tested included ampicillin/sulbactam, piperacillin/tazobactam, ceftazidime, ceftriaxone, cefepime, imipenem, meropenem, amikacin, gentamicin, ciprofloxacin, tigecycline, and colistin. American Type Culture Collection (ATCC®) *Escherichia coli* ATCC® 25922 and *Pseudomonas aeruginosa* ATCC® 27853 isolates were used as quality controls. Susceptibility was interpreted according to the CLSI^9^. The results are presented as frequency (percentage) to summarize the percentage of*A. baumannii*colonization and infection prevalence in clinical cultures, comorbidities, and outcomes among the ICUs observed in this study. Data were stored and analyzed using MS Excel XP® (Microsoft ®, Northampton, MA, USA).

In this study, 409 *Acinetobacter spp*. were identified in patients of a tertiary hospital in Dourados/MS, Brazil during the study period. Of these, 53.5% (219/409) were identified as *A. baumannii*, among which 70.8% (n = 155/219) were isolated from patients admitted to ICUs, 42.6% (n = 66/155) were classified as missing, and 57.4% (n = 89/155) were included. In addition, 92.1% (n = 82/89) of these isolates were resistant to carbapenems (Table 1) and were analyzed for infectivity and lethality rates. In August and October 2015, an increased peak of 41.6% (n = 37/82) of CRAB isolates was observed, followed by 33.7% (n = 30/82) in December 2017 and 24.7% (n = 22/82) in October 2016. CRAB isolates were most frequently isolated from males (58.5%, n = 48/82) and patients over 60 years of age (52.4%, n = 43/82). Adult ICU patients had a higher rate of CRAB (93.9%, n = 77/82). In addition, 47.6% (n = 39/82) of the CRAB isolates were isolated from tracheal aspirates, 31.7% (n = 26/82) from swabs, 8.6% (n = 7/82) from catheter tips, and 7.3% (n = 6/82) from blood cultures (Table 1). Among the patients, 63.4% (n = 52/82) had pneumonia, 43.9% (n = 36/82) had systemic arterial hypertension, 87.8% (n = 72/82) used delayed nasoenteric/bladder catheters, and 86.6% (n = 71/82) used invasive devices. 

Regarding antimicrobial susceptibility, all CRAB isolates (n = 82) showed resistance to piperacillin/tazobactam (MIC50, ≥128 mg/L), cefepime (MIC50, ≥16 mg/L), and ciprofloxacin (MIC50, ≥4 mg/L); 98.8% (n = 81/82) to ceftriaxone (MIC50, ≥32 mg/L); 97.6% (n = 80/82) to ceftazidime (MIC50, ≥32 mg/L) and imipenem (MIC50, ≥8 mg/L); 93.9% (n = 77/82) to meropenem (MIC50, ≥8 mg/L); 90% (n = 74/82) to amikacin (MIC50, ≥32 mg/L^-1^); 87.8% (n = 72/82) to gentamicin (MIC50, ≥16 mg/L^-1^); and 15.9% (n = 13/82) to tigecycline (MIC50, ≥4 mg/L^-1^). However, all the isolates (n = 82) were susceptible to colistin (MIC50, ≥2 mg/L). In this study, 40% (n = 33/82) of CRAB isolates were considered colonized, whereas 60% (n = 49/82) were infected. Unfortunately, 39 patients died, representing a mortality rate of 79.6%. Data from adult ICUs showed endemic levels in August 2015, October 2015, and December 2017 (Figure 2). The infectivity plots for neonatal and pediatric ICUs were not elaborated because the number of patients admitted was insufficient.

**FIGURE 2:**
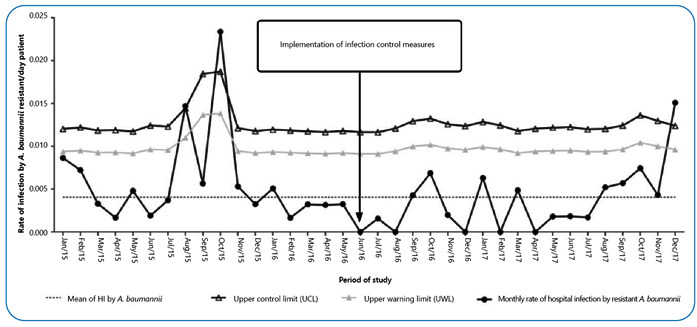
Endemic level of colonization/infection due to carbapenem-resistant *Acinetobacter baumannii* isolates per 1000 patient-days from January 2015 to December 2017. Upper control limit (3σ + X); upper alert limit (2σ+ X); X: centerline (average rate of carbapenem-resistant *A. baumannii* per 1000 patient-days); 1, adult ICU.

Following the increase in endemic levels, measures to control the spread of MDR isolates were implemented, and a reduction in the incidence of CRAB was observed. In contrast, an increase in the endemic level was reported in December of the same year (Figure 2). Thus, despite the interventions, CRAB continued to spread. A similar result was reported previously for an outbreak of colistin-susceptible CRAB. This could be explained as overuse of antibiotics, and selective preassure was considered a cause for this scenario^9^. Although colistin could be a treatment option, the development and spread of resistant isolates in the hospital needed to be considered; thus, colistin should only be used as a last resort treatment. 

MDR *A. baumannii* is considered a global problem owing to its dispersion and a challenge for hospital infection control services because of limited treatment options. The results of this study indicate a high prevalence of CRAB in ICUs. Risk factors such as prolonged hospitalization in ICUs, immunocompromised patients in critical condition, and invasive procedures favor the development of infection and a high prevalence of opportunistic bacteria such as *A. baumannii* in health facilities^10^. Following the national criteria for reporting healthcare-associated infections (HCAIs) defined by Agência Nacional de Vigilância Sanitária (ANVISA), which considers only cases that developed while the patient was in the ICU, we reported 59.75% (n = 49/82) cases in addition to the high prevalence of CRAB in ICU patients^10^. Usually, antibiotics are used extensively in developing countries, particularly in ICUs, resulting in a higher incidence of MDR and extensively drug-resistant bacteria combined with greater dissemination of these microorganisms^11^. Multidrug resistance is defined as non-susceptibility to at least one agent in three or more antimicrobial categories, whereas extensive drug resistance is defined as non-susceptibility to at least one agent in all but two or fewer antimicrobial categories (being susceptible to one or two categories)^12^. This is explained in part by the lack of resources and failure to implement control practices and prevention^13^. Moreover, comorbidities, such as pneumonia associated with mechanical ventilation, systemic arterial hypertension, diabetes mellitus, and renal failure, are associated with an increased risk of *A. baumannii* infection^13^. 

In 2017, the distribution of carbapenem-resistant *Acinetobacter* spp. in blood infections in Brazil was 77.7% in adult ICUs, 48.6% in pediatric ICUs, and 33.3% in neonatal ICUs^14^. In our study, the CRAB isolates showed 100% resistance to piperacillin/tazobactam and ciprofloxacin. More than 95% of the isolates showed resistance to third-generation cephalosporins and carbapenems, and approximately 90% were resistant to aminoglycosides. The overuse of carbapenems in the hospital environment has been reported as a leading risk factor for increased bacterial resistance. Although some studies have reported a growing increase in colistin-resistant *A. baumannii* isolates, none were found in this study^15^. 

In this study, CRAB isolates were more frequent in male patients (58.5%) and patients older than 60 years (52.4%)^15^. Although it is difficult to attribute the mortality rate to an underlying illness or the infection itself, we observed a high mortality rate associated with *A. baumannii* infection. Although the acquisition of CRAB isolates plays an important role in the high mortality rates, they are not the only risk factors responsible for the poor outcomes observed because the patients displayed several unfavorable clinical conditions^15^.

Furthermore, an increase in CRAB incidence was observed in August 2015, October 2015, and December 2017. Stringent infection control measures were introduced to prevent further spread, and the outbreak was declared to be under control in November 2015. However, our data showed that the control measures might have had a short-term effect, as they gradually lost efficacy in the following months. The number of new acquisitions of CRAB isolates started to increase in the ICUs, reaching the alert threshold in December 2017. Although active surveillance contributed to reducing the rates of this pathogen through control measures, our results highlighted the difficulty in eradicating this pathogen, indicating the possibility of selection and spread of clonal carbapenem-resistant isolates at a certain point. Additionally, control and containment of outbreaks are essential to reduce the impact of CRAB in the hospital environment. 

However, the identification of isolates in this study was not confirmed by other techniques, such as MALDI-TOF and sequencing. We also highlight the importance of multicenter studies in Brazil for better comparison, comprehension, and validation of specific interventions in each hospital. Despite these limitations, this study showed a significant prevalence of CRAB isolates in a Brazilian hospital associated with a high mortality rate and difficulty in controlling the spread of CRAB in hospital wards. Epidemiological research may help monitor the dispersion of these isolates and contribute to the development of efficient infection control policies and containment intervention programs for this pathogen.
